# Physiological Impairment as a Result of Bile Accumulation in an Apex Predator, the Tiger Shark (*Galeocerdo cuvier* Péron & Lesueur, 1822)

**DOI:** 10.3390/ani10112030

**Published:** 2020-11-04

**Authors:** Natascha Wosnick, Ana Paula Chaves, Yuri Vieira Niella, Veronica Takatsuka, Fábio Hissa Vieira Hazin, Jorge Luiz Silva Nunes, Danny Morick

**Affiliations:** 1 Programa de Pós-graduação em Zoologia, Universidade Federal do Paraná, Curitiba 81530-000, Brazil; 2 Laboratório de Organismos Aquáticos, Universidade Federal do Maranhão, São Luís 65080-805, Brazil; apaula.cs19@gmail.com (A.P.C.); silvanunes@yahoo.com (J.L.S.N.); 3 Department of Biological Sciences, Macquarie University, Macquarie Park, Sydney, NSW 2109, Australia; yuri.niella@gmail.com; 4 Instituto Pró Peixes, Ubatuba 11680-000, Brazil; veronicatakatsuka@gmail.com; 5 Laboratório de Biologia Pesqueira, Universidade Federal Rural de Pernambuco, Recife 52171-900, Brazil; fhvhazin@terra.com.br; 6 Morris Kahn Marine Research Station, University of Haifa, Haifa 3498838, Israel; dmorick@univ.haifa.ac.il; 7 Hong Kong Branch of Southern Marine Science and Engineering Guangdong Laboratory, Guangzhou 511458, China

**Keywords:** gallbladder, hepatobiliary alteration, physiological markers

## Abstract

**Simple Summary:**

Sharks rely on a diet with high lipid content, depending on gallbladder proper functioning for lipid emulsification, absorption, and subsequent hepatic storage. The present study reports a physiological impairment due to bile accumulation in juvenile tiger sharks and the possible causes of such condition.

**Abstract:**

Physiological adaptations have evolved to help sharks face rapid periods of feast. Tiger sharks are generalist apex predators that rely on a high-lipid/protein diet. To achieve a satisfactory nutritional condition, proper lipid absorption and hepatic storage are needed. Bile secretion in sharks is low and sporadic but increases during short periods of fasting. The present study describes a physiological impairment caused by bile accumulation in juvenile tiger sharks, possibly due to prolonged fasting. These evidences suggest that, even though sharks have adaptations that prevent them from dying from starvation, alarming physiological alterations might occur. Future studies are needed to assess how such a condition can affect wild populations, as well as possible sublethal consequences that could impact their long-term survival.

## 1. Introduction

Sharks are one of the most endangered groups of vertebrates, with population declines of up to 90% for some species in some regions [1]. In addition to overexploitation, the reduction of genetic variability is of particular concern as it compromises phenotypic plasticity and adaptations in response to environmental changes and stressors. Habitat loss and pollution are emerging threats to marine animals, leading to increased incidences of pathologies in wild populations [2]. Many diseases, although not lethal, may compromise physical and nutritional conditions, thus affecting fitness and subsequent population recruitment. Reports of diseases in sharks and batoids (Elasmobranchii) are mainly from captive animals. In a 17-year survey comprising a total of 1546 cases, infectious/inflammatory diseases were the most observed pathology types [3]. Despite the recent advances in shark diagnostics, liver diseases have as yet been little explored. Liver neoplasms have been described for sharks, including hepatic adenoma [4], hepatic capsular fibroma [5], and hepatic cholangiocarcinoma [6]. For the biliary system, our knowledge is even more incipient, with diagnoses limited to parasitic diseases available in the literature [3].

On vertebrates, physiological alterations often occur as direct effects of hepatobiliary dysfunctions, such as elevated activities of Alkaline Phosphatase (ALP), Alanine Transaminase (ALT), and Gamma-Glutamyl Transferase (GGT) [7]. ALP is a physiological marker widely used in vertebrate studies and plays an important role in liver metabolism [8], with elevated levels being indicative of alterations in gallbladder/liver general functioning [7]. It is also commonly used to access liver integrity, with increased activity being observed when there is damage to the hepatocyte membranes, leading to higher permeability [9]. ALT is also a reliable marker to access liver disorders, with elevated circulating levels observed in severe hepatocellular disease in fish [10]. GGT is present in the membranes of hepatic cells and is responsible for maintaining intracellular homeostasis from oxidative stress. Elevated serum GGT is also observed in liver, biliary system, and pancreas diseases [7]. Bilirubin (BR) is also evaluated when hepatobiliary alteration is suspected, with elevated levels possibly as an indicator of impaired uptake, conjugation, or excretion at hepatocytes or damaged/obstructed bile ducts, resulting in blocked flow [11]. Lipid dynamics can also be affected by liver disease with increased levels of circulating triglycerides and total cholesterol [7]. To date, the use of plasma markers that indicate structural and functional hepatobiliary damage in sharks is restricted to studies of capture stress or simply presented as reference intervals [12,13,14,15,16]. Moreover, although widely used as diagnostic tools, physiological markers have only been evaluated for a few shark species.

The tiger shark, *Galeocerdo cuvier,* is a generalist apex predator and also an efficient scavenger commonly associated with coastal and insular shelves [17,18]. Tiger shark life cycle is characterized by late maturity, long life span (up to 50 years), and long gestation periods (14–16 months) [19]. The species has an exclusive reproductive strategy called ‘embryotrophy’, which consists in an adaptation for large-scale production of embryos without placental connection [19]. Intrinsic features of its life cycle, coupled with its dwelling in areas extensively used by humans, such as urbanized coastal habitats, make them more vulnerable to anthropogenic impacts [20]. Seasonal use of coastal areas [21,22] is related to prey abundance [22,23]. Despite some individuals showing strong site fidelity, others might perform transoceanic migrations [24,25], responsible for large home ranges over variable temporal scales [26]. Considering its feeding habits, reproductive mode, and migratory behavior, the tiger shark is a promising study model to assess the relationships between physiological traits, metabolic dynamics, and ecology [27].

Due to the absence of adipose tissue in sharks, the liver is the main organ responsible for lipid storage [28]. Furthermore, the liver plays an important role in lipid metabolism during pregnancy and migration [28]. Recent studies indicated that higher concentrations of circulating triglycerides are related to better body condition in tiger sharks [27]. However, no relationship between circulating triglycerides and gestational period has been observed for the species, indicating that other lipid sources may be used during embryonic development [28]. In juveniles, circulating levels of total cholesterol appear to be related to movement patterns, with higher plasma concentrations observed in tiger sharks that undertook migrations [29]. By monitoring the landing of artisanal fisheries in the northeast coast of Brazil, our team had access to two juvenile female tiger sharks recently deceased showing an unusual bile accumulation in the gallbladder and hepatobiliary ducts. To date, there were no reports of hepatobiliary alterations in sharks, nor investigations on its potential correlations with physiological alterations indicative of functional impairment. To shed light on this matter, the present study aimed to evaluate the effects of the observed alterations upon physiological markers traditionally used to access hepatobiliary and nutritional disorders in vertebrates. Moreover, taking into account that post-mortem alterations in the chosen markers are expected to occur, data from both deceased juvenile females with bile accumulation were compared with data from other dead juveniles without any visible alterations and data from alive and released juvenile tiger sharks.

Here, we hypothesize that significant alterations in circulating levels of the chosen physiological markers would be observed in tiger sharks and related to the observed bile accumulation. In addition, significant differences would be observed between the biological groups tested, possibly related to variable levels of ALP, ALT, and GGT in the females with bile accumulation. Finally, alterations in circulating bilirubin and serum lipid concentrations are expected to be higher in the sharks with bile accumulation due to hepatobiliary disorder.

## 2. Materials and Methods

### 2.1. Tiger Sharks

**Figure 1:**
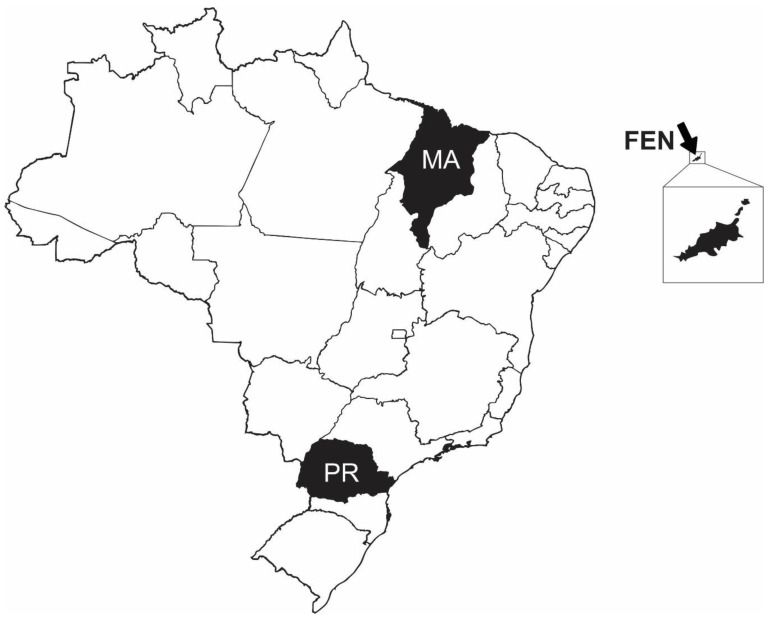
Sampling sites. MA—Maranhão State, northeastern Brazil, location where tiger sharks with bile accumulation were caught. FEN—Fernando de Noronha Archipelago, northeastern Brazil, location where sharks were captured and released during scientific campaigns. PR—Paraná State, southern Brazil, location where sharks used as putative control (post-mortem) were caught.

Two juvenile females with bile accumulation were captured by the artisanal fleet in the State of Maranhão, northeastern Brazilian coast (Figure 1). The animals were caught in September 2019 using a 5 km longline equipped with 100 circular hooks (14/0) baited with hake (*Merluccius* spp.). Both sharks were caught at the continental shelf, 15 km from the coast and 50 m depth in fishing campaigns carried out between 4 and 9 am. The longline stayed in the water for 5 h, however, it was not possible to estimate the exact time that the animals were hooked. After capture, the sharks were brought on board and kept refrigerated on ice until landing at the fish market (~1 h). Approximately 30 min after unloading, the blood samples were collected and necropsy performed, thus totaling less than 7 h between capture and sampling. Sampling was approved by the Brazilian Ministry of Environment (IBAMA/ICMBio-SISBIO #60306-1).

For comparative purposes, dead juvenile tiger sharks with no visible hepatobiliary alterations (*n* = 6) captured as bycatch from artisanal fisheries in the state of Paraná, southern Brazil (Figure 1), were also sampled. These animals were caught between December 2013 and March 2015, with gillnets (10 cm mesh). All sharks were caught along the continental shelf, between 25 and 40 km from the coast, and during fishing campaigns conducted between 6 am and 4 pm. The fishing campaigns lasted for about 10 h and the gillnet soak times were around 6 h. Again, it was not possible to determine the exact time that sharks were caught. After capture, the animals were brought on board and transported to the landing site (~2 h) still trapped in the net. Upon arrival, the sharks were removed from the gillnet and the blood samples were then taken, approximately 1 h after landing. Therefore, all these samples were collected within less than 12 h after capture. Sampling was approved by the Brazilian Ministry of Environment (IBAMA/ICMBio-SISBIO #70982).

For dead sharks, a freshness index was applied, considering the following variables: (1) overall color of the gills, (2) level of ocular retraction, (3) blood clotting, and (4) rigor-mortis. Only sharks with a reddish color in the gills, without ocular retraction, without blood coagulation, and without rigor-mortis were sampled. Some sharks presented bruises likely due to internal bleeding caused by fighting in the fishing apparatus. Moreover, ice burns were observed in the individuals with bile accumulation due to the storage process adopted by the fishermen.

Data from live tiger sharks (*n* = 5) captured in the Fernando de Noronha Archipelago, northeastern Brazil (Figure 1), were also included. The sharks were captured around the Archipelago using handlines equipped with circle hooks (17/0) baited with sardines (*Sardinops* spp.), at depths ranging from 30 to 40 m. All sharks were caught in fishing campaigns carried out between 6 am and 6 pm in July 2014. Since these were dedicated scientific campaigns, sharks were caught individually and blood samples collected only about 12 min after hooking. Sampling was approved by the Brazilian Ministry of Environment (IBAMA/ICMBio-SISBIO #15083-8) and by the Ethics Committee on Animal Use (23082.003679/2009—UFRPE).

### 2.2. Blood Sampling

Following body measurements (Table 1), blood samples (10 mL) were obtained by caudal venipuncture using a 18 G needle attached to a 20 mL disposable syringe, and immediately transferred to ultra-pure polypropylene microcentrifuge tubes (2 mL) (Tubes^®^ 3810X, Eppendorf-Hamburg, Germany). Blood sampling followed the standardized protocol for sharks [30,31]. The blood samples collected in Maranhão and Paraná (dead tiger sharks) were immediately stored in a polystyrene box with ice and taken to the Universities (Federal University of Maranhão UFMA—30 km away from the collection site, and Federal University of Paraná UFPR—100 km away from the collection site). Upon arrival, the blood samples were centrifuged for 7 min at room temperature (20 °C) at 2000 g. Serum was separated and kept frozen at −20 °C until analysis at the Food and Water Quality Control Laboratory (UFMA) and at the Laboratory of Comparative Physiology of Osmoregulation (UFPR). The samples collected in Fernando de Noronha were immediately centrifuged on board the M/V Ocearch (7 min at room temperature at 2000 g) and serum was stored frozen at −20 °C. For transportation by plane to the Federal University of Paraná (~15 h), these samples were stored in a polystyrene box with dry ice (−78 °C). Upon arrival, samples were kept in an ultra-freezer (−82.5 °C) until they were analyzed. All serum samples had a color ranging from transparent (live sharks) to light pink (dead sharks), indicating mild hemolysis.

### 2.3. Serum Assays

To test liver function and integrity, the activity of the enzymes ALP, ALT, and GGT was evaluated for all sharks, whereas bilirubin (BR) levels were used to evaluate gallbladder functioning. The possible impacts of bile accumulation in the nutritional status of sharks were investigated using triglycerides and total cholesterol in the serum. Circulating ALP (Labtest–Brazil; catalog n. 40 wave-length 590 nm), ALT (catalog n. 108; wave-length 340 nm), GGT (Labtest–Brazil; catalog n. 105 wave-length 405 nm), BR (catalog n. 31; wave-length 525 nm), triglycerides (Labtest–Brazil; catalog n. 87; wave-length 505 nm), and total cholesterol (Labtest–Brazil; catalog n. 76; wave-length 500 nm) were quantified colorimetrically (Visible UV Spectrophotometer Q898U2M5 Quimis, Diadema, Brazil) All assays were carried out strictly following the manufacturer’s instructions and with previously sterilized material to avoid contamination. Means of each marker for each biological group (LS, DS, and SBA) are presented as supplementary material (Table S1).

### 2.4. Liver and Gallbladder Histopathology

Samples of the liver and gallbladder of the shark SBA2 were collected and kept frozen at −80 °C. Two fragments from six distinct regions of the liver and two fragments of the gallbladder were sectioned (1 cm^3^ each) and fixed in 10% formaldehyde. The samples were sent to the Veterinary Anatomopathological Analysis laboratory (HistoPato; Federal District, Brazil) for histopathological evaluation. Upon arrival, the samples were dehydrated in an increasing series of alcohol content (ethanol—70% to 100%), diaphanized in xylol, and embedded in paraffin. Samples were then sectioned at 5 μm, stained with hematoxylin-eosin (HE), and placed on glass slides using standard histological techniques.

### 2.5. Statistical Analysis

To assess the overall relationships between the physiological markers and the observed conditions, a Principal Component Analysis (PCA) was used including SBA, DS, and LS groups. One-way analysis of variance (ANOVA) with post-hoc of Holm–Sidak was used to assess differences in the activities of the enzymes ALP, ALT, and GGT and in serum concentrations of BR, triglycerides, and total cholesterol among sharks with bile accumulation (SBA), dead sharks without visible morphological alterations (DS), and live sharks (LS). Lastly, to assess the effects of bile accumulation upon the nutritional status of tiger sharks, the condition proxy (ratio of TAG:CHOL) of each shark was calculated and again compared between the groups (SBA, DS, and LS) using a one-way ANOVA. All tests were performed using R software (R Development Core Team 2016) with a limit of significance of 0.05.

## 3. Results

### 3.1. Sharks with Bile Accumulation Disorder

**Figure 2:**
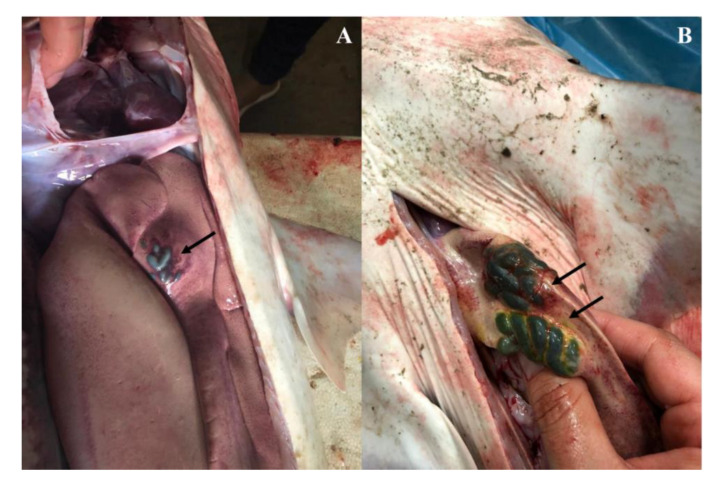
Clusters of bile-filled cysts in the left lobe of the liver of two juvenile female tiger sharks. (**A**) Photographic record of juvenile female (SBA1) liver. (**B**) Photographic record of juvenile female (SBA2) liver. Black arrows indicate bile-filled cysts.

In sharks with accumulated bile, clusters of bile-filled cysts were detected. In female 1 (SBA1), fewer cysts were observed ranging from 0.5 to 1 cm (Figure 2A), whereas female 2 (SBA2) had two clusters of green cysts with size ranging between 1 and 1.5 cm (Figure 2B). In both females, the cysts were located in the anterior portion of the left liver lobe. No other visual alterations were observed in these individuals. During necropsy, the stomach and intestine of both females were analyzed for the presence of food remains. No stomach or intestinal contents were found, possibly indicating that both had not fed recently. Furthermore, since shark weight indicated by the fishermen’s scale was not compatible with that expected for their size class, this metric was not considered, preventing us from calculating Fulton’s condition factor and hepatosomatic index for the dead sharks (SBA and DS).

**Figure 3:**
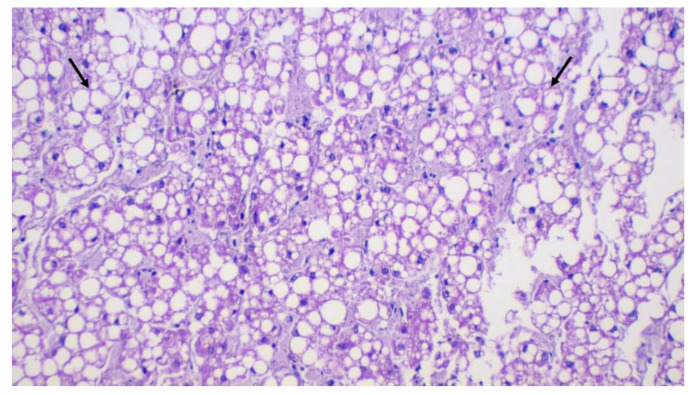
Histopathological analysis of the liver of a tiger shark with bile accumulation (SBA2). Black arrows indicate hepatocytes filled by large intracytoplasmic vacuoles. H&E, 20× increase.

Histopathological analyses performed on hepatic fragments of the shark SBA2 indicated hepatocytes filled with large vacuoles, with a displacement of the nucleus to the periphery, a histological arrangement commonly observed in sharks due to their high lipid liver content [32]. Lymphocytes and plasma cells were detected in the periportal region and inside sinusoids (Figure 3). In addition, mild inflammation around the hepatobiliary ducts was detected. No infectious agents were observed. Due to accentuated gallbladder autolysis, it was not possible to perform histopathological analysis, preventing us from further detecting possible structural and functional alterations.

### 3.2. Overall Distribution of Biomarker Concentrations

**Figure 4:**
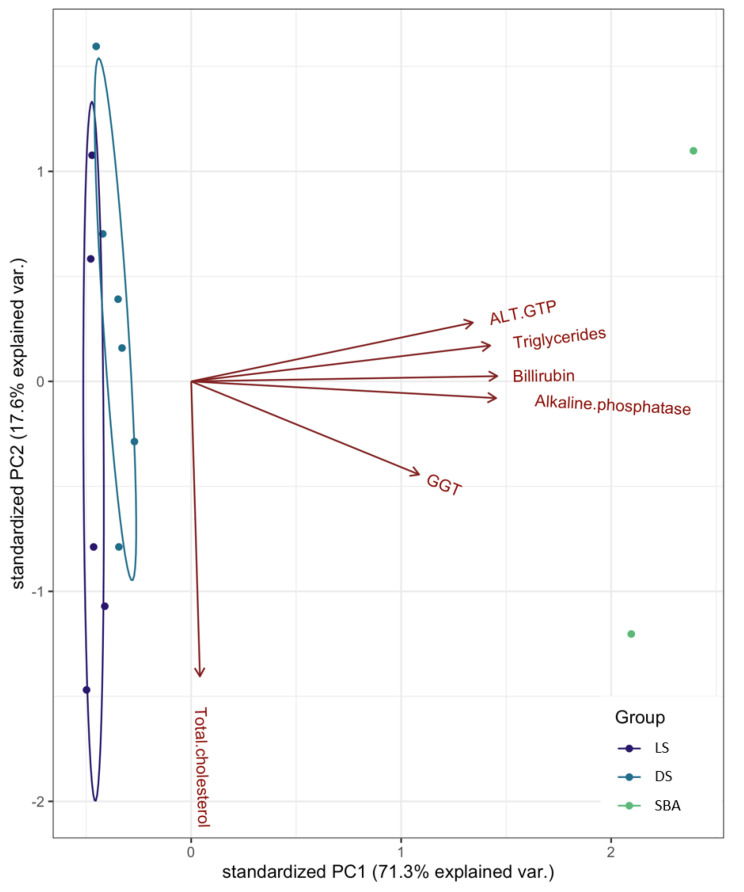
Principal component analysis (PCA) biplot of biomarker concentrations as a function of shark groups (LS—live sharks, DS—dead sharks, SBA—sharks with bile accumulation).

The PCA analysis (total variance explained = 88.9%) indicated some similarity between the physiological markers and two of the conditions observed (i.e., live sharks and dead sharks with no bile accumulation). The majority of the variability in the data was explained by the X axis (71.3% explained variance) and was driven by most of the biomarkers, besides total cholesterol (17.6%) (Figure 4). Total cholesterol was highly variable within groups, even for the sharks with bile accumulation (SBA), which included only two specimens. Activities and concentrations of the remaining biomarkers were lower in live sharks, slightly higher in dead sharks, and significantly more elevated in sharks with bile accumulation (Figure 4).

### 3.3. Enzyme Activities and Circulating Bilirubin

**Figure 5:**
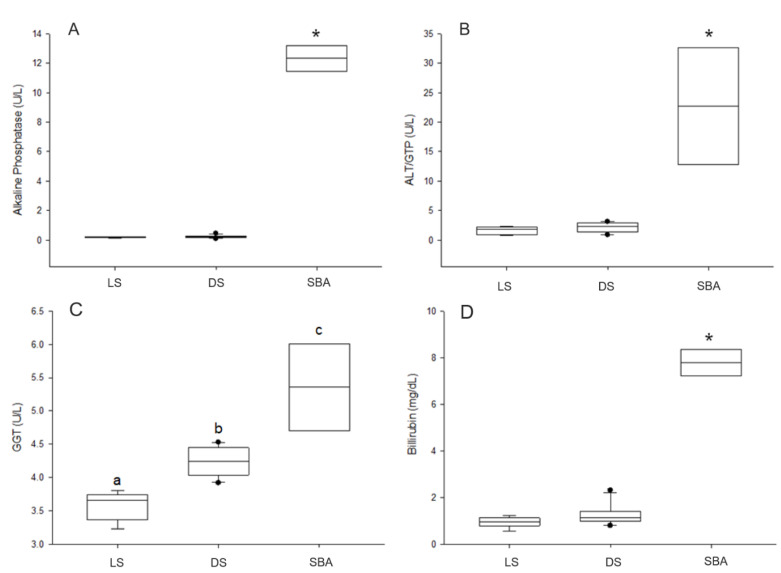
Hepatobiliary markers analyzed in tiger sharks. (**A**) Alkaline Phosphatase (ALP) activities, (**B**) Alanine Transaminase (ALT) activities, (**C**) Gamma-Glutamyl Transferase (GGT) activities, (**D**) Bilirubin concentrations. LS—live sharks, DS—dead sharks, SBA—sharks with bile accumulation. Boxplots respectively represent minimum, first quarter, median, third quarter, and maximum values. Post-hoc groupings are represented by lower case letters and (*).

Significant differences in ALP activity were observed in sharks with bile accumulation when compared to the other shark groups (*p* < 0.001) (Figure 5A). However, there was no difference in enzyme activity between dead sharks without visible alterations and live sharks (*p* = 0.826). As for ALT, differences in activity were also observed in sharks with bile accumulation when compared to the other groups (*p* < 0.001) but there was no difference between dead sharks without alterations and live sharks (*p* = 0.854) (Figure 5B). In contrast, GGT activity was significantly different between groups (*p* = 0.008), being highest in sharks with bile accumulation, followed by dead sharks without visible alterations (Figure 5C). Significant differences in circulating bilirubin were also observed in sharks with bile accumulation when compared to other sharks (*p* < 0.001) (Figure 5D). No difference was detected in serum concentrations between dead sharks without visible alterations and live sharks (*p* = 0.263).

### 3.4. Nutritional Profiles

**Figure 6:**
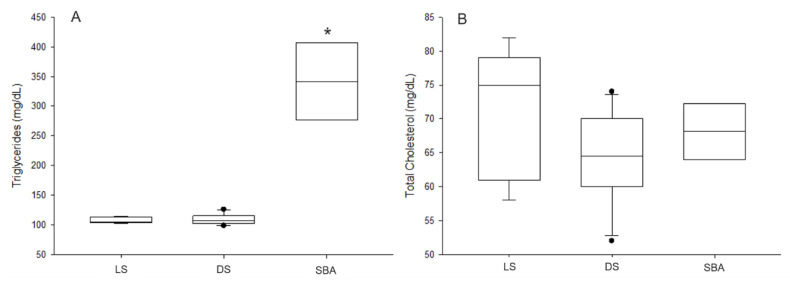
Metabolic markers analyzed in tiger sharks. (**A**) Triglycerides concentrations, (**B**) Total cholesterol concentrations. LS—live sharks, DS—dead sharks, SBA—sharks with bile accumulation. Boxplots respectively represent minimum, first quarter, median, third quarter, and maximum values. Statistical differences are represented by (*).

**Figure 7:**
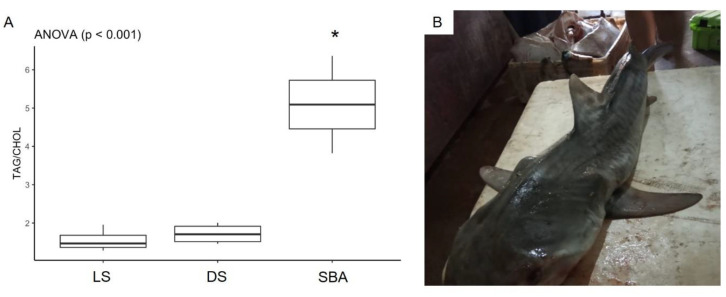
(**A**) Condition proxy of tiger sharks analyzed in the present study. LS—live sharks, DS—dead sharks, SBA—sharks with bile accumulation. Boxplots respectively represent minimum, first quarter, median, third quarter, and maximum values. Statistical differences are represented by (*). (**B**) Photographic record of female with bile accumulation (SBA1) evidencing its thinness.

Significantly higher concentrations of serum triglycerides were observed in sharks with bile accumulation in relation to the other groups (DS and LS) (*p* < 0.001) (Figure 6A). However, there was no difference in concentrations between dead sharks without visible alterations and live sharks (*p* = 0.949). On the other hand, no significant differences were observed between groups for serum total cholesterol concentrations (*p* = 0.461) (Figure 6B). Regarding condition proxy (ratio of TAG:CHOL), the results showed significant differences for sharks with suspected pathology when compared to the other shark groups (*p* < 0.001) (Figure 7A).

## 4. Discussion

The tiger shark is a generalist apex predator and a promising model to evaluate morphophysiological alterations linked to emerging pathologies or nutritional disorders, and how such conditions may affect high trophic level animals. Although the morphological alterations observed in the histopathological analysis were discreet, there is strong evidence of physiological impairment caused by the accumulation of bile observed. To date, all investigations on shark bile aimed to assess composition [33,34,35,36,37], pigment concentrations (i.e., biliverdin and bilirubin) [38,39,40], molecular pathways involved in synthesis [40,41,42], and the biliary elimination of toxic compounds [43,44]. According to previous studies, the secretion of bile in sharks ranges from 0.5 to 2.8 mL/kg/24 h [43], which is considered low, being around 100 times lower than in rodents, for instance [38]. Besides, bile can be quickly deteriorated as a consequence of confinement conditions. Such a condition can be fatal as it leads to a significant reduction in hepatic and renal flow and in blood pH [45].

Increased bile secretion was observed as a result of fasting in the dogfish shark (*Squalus acanthias*) [45,46]. However, the possible causes of such an increase were not explored by the authors. In the present study, both juvenile females showed a significant bile retention within the gallbladder and the hepatobiliary ducts. A similar condition has already been observed in a newborn tiger shark fasting for more than five days (Veronica Takatsuka, personal observation) and wild adult *Carcharhinus obscurus* (Danny Morick, personal observation). No stomach content or feces were found in the females that showed bile accumulation. Also, both sharks were very thin (Figure 7B—female SBA1), suggesting that both were likely experiencing periods of prolonged fasting. Moreover, mild cholestasis could not be ruled out, as the accumulation observed may be related to the inability to properly secrete rather than increased production. Besides from the impact of reduced nutrient absorption (e.g., calcium and vitamin D) due to the interruption of bile flow to the duodenum, bile retention in hepatobiliary ducts is also problematic due to its toxicity [47].

Considering the physiological markers tested, we found partial support for our first hypothesis that significant alterations in the circulating levels of the physiological markers would be observed in tiger sharks with bile accumulation. When evaluated separately, the chosen markers may indicate alterations not necessarily linked to the hepatobiliary system (e.g., strenuous exercise) [12]. However, considering that all markers, apart from total cholesterol, exhibited higher activities and concentrations in sharks with bile accumulation, it is plausible that the observed physiological impairment is likely related to this condition. However, a proper evaluation of systemic health and emerging pathologies is hindered by the challenges in keeping tiger sharks in captivity. Consequently, the possible causes and consequences of the condition reported in the present study cannot be inferred.

Consistently with our second hypothesis, we found elevated activities of ALP, ATL, and GGT in females with bile accumulation. Activities were higher not only when compared to live sharks, but also in relation to dead sharks with no visual morphological alterations. That said, even when post-mortem artifacts are in place [48], significant alterations in the physiological response of sharks presenting bile accumulation are observed. Despite the possible influence of capture stress on the activity of the analyzed enzymes, there is strong evidence that the patterns observed are mainly due to the accumulation of bile, since the dead tiger sharks accessed for comparative purposes exhibited lower ALP and ALT activities that did not differ from live sharks. GGT activity was significantly higher in dead sharks independent of hepatobiliary alterations (SBA and DS) when compared to live animals (LS). This pattern indicates that GGT is more sensitive to post-mortem artifacts and therefore, should be interpreted with caution.

We found empirical evidence that supports our third hypothesis, that bilirubin concentration would be higher in sharks with bile accumulation. Such an increase was expected as pathologies associated with hepatobiliary ducts’ obstruction or inability to properly secrete bile can lead to pigment leak into the bloodstream [8]. In elasmobranch bile, there is a predominance of biliverdin over bilirubin, and both significantly increase during prolonged fasting and confinement periods [46]. That said, serum concentrations of bilirubin in the sharks with bile accumulation are another strong evidence of prolonged fasting leading to physiological alterations.

Lastly, we found mixed evidence for our fourth hypothesis of an expected increase in serum lipid concentrations in sharks with hepatobiliary alterations. While concentrations of triglycerides did not differ significantly between live and dead sharks without visible alterations, concentrations were markedly higher in juvenile females with bile accumulation. Since sharks strongly rely on lipid metabolism for energy intake [49], the high circulating levels observed could be a result of hepatic reserves assimilation as a means to compensate for prolonged fasting. Unlike what was expected, total cholesterol serum concentrations did not differ between shark groups, being highly variable among individuals. Total cholesterol plays an important role in hormonal synthesis [50], thus the observed pattern may be related to other physiological pathways rather than digestive activity modulated by the hepatobiliary system. The ratio between TAG and CHOL as a nutritional proxy has recently been validated for sharks to assess energy metabolism [51]. It is expected that sharks in better nutritional conditions would exhibit higher ratios. Our results refute this theory as sharks with physiological impairment due to bile accumulation were in fact the ones that presented the highest TAG:CHOL ratios. Caution is needed when interpreting nutritional condition based solely on this proxy, since metabolic disruptions can significantly alter circulating lipid levels.

## 5. Conclusions

The accumulation of bile observed in the present study led to a series of physiological disruptions that can significantly compromise the health of apex predators that rely on a high-lipid diet such as sharks [52] and, therefore, depend on proper secretion of bile salts for lipid emulsification. Although it was not possible to confirm a diagnosis, the results indicate that prolonged fasting may have been the main cause of the accumulation observed. Tiger sharks exhibit ontogenetic feeding shifts, with juveniles preying mainly on teleost fish and cephalopods [53]. Due to their more generalist behavior, tiger sharks usually target a wide variety of prey items. Moreover, as scavengers, their feeding opportunities are even greater [53,54]. These animals tend to ingest large meals at irregular intervals, going days or weeks without feeding again [52]. Besides, there is evidence that predatory fish have evolutive adaptations that modulate physiological responses to fasting, which reduces the metabolic impacts during periods of prey scarcity [55]. Considering that tiger sharks have sporadic feeding opportunities and that sharks are evolutionarily adapted to fasting for weeks, it is plausible to infer that both juvenile females with bile accumulation and evidence of physiological impairment analyzed in the present study were under severe feeding restrictions which may be related to pathologies not yet investigated in this group of animals. Despite the evident thinness, hepatic lipid reserves were relatively well preserved, indicating that during severe fasting, the hepatic stores might prevent sharks from dying of starvation, even when under physiological disruption conditions.

## Figures and Tables

**Table 1:** Tiger sharks analyzed in the present study. Sex, life stage and total length (TL), fishing method, and fishing location are presented for each shark. SBA—sharks with bile accumulation, DS—dead sharks, LS—live sharks.

Tiger Sharks	Sex	Stage	TL (cm)	Fishing Method	Location
SBA 1	Female	Juvenile	170	Longline	MA
SBA 2	Female	Juvenile	172	Longline	MA
DS 1	Female	Juvenile	175	Gillnet	PR
DS 2	Female	Juvenile	210	Gillnet	PR
DS 3	Female	Juvenile	180	Longline	PR
DS 4	Male	Juvenile	135	Longline	PR
DS 5	Male	Juvenile	148	Longline	PR
DS 6	Male	Juvenile	155	Gillnet	PR
LS 1	Female	Juvenile	259	Handline	FEN
LS 2	Male	Juvenile	182	Handline	FEN
LS 3	Male	Juvenile	204	Handline	FEN
LS 4	Male	Juvenile	244	Handline	FEN
LS 5	Female	Juvenile	243	Handline	FEN

## Supplementary Materials

The following are available online at https://www.mdpi.com/2076-2615/10/11/2030/s1, Table S1: Serum physiological markers analyzed in the present study.
