# Supplementary material for: Physiological Impairment as a Result of Bile Accumulation in an Apex Predator, the Tiger Shark (Galeocerdo cuvier Péron & Lesueur, 1822)
Source: Animals (Basel). 2020 Nov 4;10(11):2030. doi: 10.3390/ani10112030 (PMC7694183; doi:10.3390/ani10112030)
Supplement: Supplementary file 1 [file animals-10-02030-s001.pdf]

Supplementary material

Table. S1. Serum physiological markers analyzed in the present study. SBA – sharks with bile accumulation; DS – dead sharks; LS – live sharks. Values are presented as mean ± S.D.

Tiger sharks	Sample size	ALP (U/L)	ALT (U/L)	GGT (U/L)	Bilirubin (mg/dL)	Triglycerides (mg/dL)	Total cholesterol (mg/dL)
SBA	2	12.32±1.25	22.7±14	259.3±210.5	7.8±0.8	341.6±92.6	68.1±5.8
DS	6	0.25±0.12	2.1±0.9	70.7±16.4	1.29±0.54	109.1±9.9	64.2±7.7
LS	5	0.19±0.04	1.6±0.7	36±8.2	0.94±0.25	107.9±5.6	71±10.4
